# Transforming growth factor-β/Smad3 signalling regulates inflammatory responses in a murine model of contact hypersensitivity

**DOI:** 10.1111/j.1365-2133.2008.08696.x

**Published:** 2008-09

**Authors:** M Anthoni, N Fyhrquist-Vanni, H Wolff, H Alenius, A Lauerma

**Affiliations:** Unit of Excellence in ImmunotoxicologyFIN-00250 Helsinki, Finland; * Team for Biological Mechanisms and Prevention of Work-Related DiseasesFIN-00250 Helsinki, Finland; § Control of Hypersensitivity Diseases, Finnish Institute of Occupational HealthFIN-00250 Helsinki, Finland; † Department of Pathology, Helsinki University Central HospitalHelsinki, Finland; ‡ Department of Pathology, Kymenlaakso Central HospitalKotka, Finland; ¶ Department of Dermatology, Venereology and Allergology, University of HelsinkiHelsinki, Finland

**Keywords:** chemokine, contact hypersensitivity, cytokine, murine model, Smad3, transforming growth factor-β

## Abstract

**Background:**

Transforming growth factor (TGF)-β is an important modulator of immune functions and cellular responses, such as differentiation, proliferation, migration and apoptosis. The Smad proteins, which are intracellular TGF-β signal transducers, mediate most actions of TGF-β.

**Objectives:**

This study examines the role of Smad3 in a murine model of contact hypersensitivity (CHS).

**Methods:**

The CHS response to oxazolone was studied in Smad3-deficient mice. The ear swelling response was measured and skin biopsies from oxazolone-sensitized skin areas were obtained for RNA isolation, immunohistochemical analyses and histology. Ear draining lymph nodes were collected for RNA isolation and proliferation tests. Quantitative real-time polymerase chain reaction was used to quantify mRNA expression of cytokines, chemokines and transcription factors.

**Results:**

The expression of proinflammatory [interleukin (IL)-1β, tumour necrosis factor-α, IL-6], Th2 (IL-4) and Th17 type cytokines (IL-17), as well as regulatory components (TGF-β, Foxp3) increased significantly at the mRNA level in the skin of oxazolone-treated Smad3−/− mice when compared with wild-type controls. The expression of the Th1 type cytokine IFN-γ and the chemokines CXCL9 and CXCL10 was, however, unaffected by the lack of Smad3. The number of neutrophils and expression of the chemokines CCL3 and CXCL5, which are both involved in neutrophil recruitment, were increased in mice lacking Smad3. Also Th2 type chemokines CCL24, CCL3 and CXCL5 were increased in the skin of Smad3−/− mice compared with wild-type mice. In the lymph nodes, mRNA of IL-1β and IL-17, but not IL-4, TGF-β or Foxp3, was increased in Smad3−/− mice during the CHS response.

**Conclusions:**

The lack of intact TGF-β signalling via Smad3 results in an increased proinflammatory, Th2 and Th17 type response in the skin, as well as increased expression of regulatory elements such as TGF-β and Foxp3. Understanding the role of Smad3 in the CHS response may offer treatment and prevention strategies in this often disabling disease.

Allergic contact dermatitis (ACD) is an inflammatory response occurring at the site of hapten challenge in sensitized individuals. It has a chronic course with relapses occurring at every contact with the allergen. ACD is a major occupational and environmental dermatological health problem, often causing work disability due to occupational exposure to contact allergens (haptens).^1^ The inflammatory infiltrate in ACD is composed of CD8+ and CD4+ T cells, dendritic cells (DCs), eosinophils, monocytes and an early and transient presence of neutrophils.^2^–^5^ Cytokines produced by T-helper (Th) 1 and Th2 type cells are involved in the expression of contact hypersensitivity (CHS).^2^,^3^,^6^,^7^

Transforming growth factor (TGF)-β is a multifunctional cytokine involved in diverse biological processes ranging from cell differentiation and tissue repair to development of tumours and inflammation. The importance of TGF-β signalling is evident from the severe immune pathology seen in TGF-β knockout mice.^8^ TGF-β is most commonly considered an anti-inflammatory cytokine, inhibiting Th1 and Th2 differentiation and the production of, and response to, cytokines associated with both subtypes.^8^,^9^ TGF-β also exerts potent inhibitory effects on macrophages and DCs, and induces the differentiation of T-regulatory cells (Tregs).^10^–^12^ Paradoxically, several proinflammatory mechanisms have also been described, including a role in mice for TGF-β in the differentiation of Th17 cells. Th17 cells, which have been linked to the pathogenesis of several inflammatory and autoimmune diseases, including CHS,^13^ require in the mouse TGF-β, interleukin (IL)-1β, IL-6 and the transcription factor RORγt for their differentiation.^14^,^15^ Furthermore, TGF-β is a strong chemoattractant for leucocytes and modulates the expression of adhesion molecules.^8^ The analysis of the role of TGF-β in the immune system is complicated by the fact that most tissues produce TGF-β and express receptors for TGF-β.

The general pathway of TGF-β signalling involves the binding of mature TGF-β1 to the TGF-β type II receptor, which in turn activates the TGF-β type I receptor (TGFβRI). Upon activation, TGFβRI phosphorylates intracellular mediators called Smad proteins, which are central in most actions of TGF-β.^16^,^17^ The Smads include receptor-specific Smads (R-Smad1, 2, 3, 5 and 8), the common Smad (Co-Smad4) and inhibitory Smads (I-Smad6 and 7). The phosphorylated R-Smads form protein complexes with Smad4 molecules which subsequently translocate into the nucleus where they regulate transcription of target genes.^17^ I-Smads are also induced by TGF-β family ligands, but terminate or reduce the strength of the signal.

The generation of mice deficient in Smads has made it possible to investigate the relative role of these mediators of TGF-β signalling *in vivo*. Unlike the targeted disruptions of Smad2 and 4 which are lethal, mice with targeted disruption of Smad3 are viable and survive to adulthood (up to approximately 8 months of age).^18^ Eventually, Smad3 knockout mice develop progressive illness including leucocytosis, massive inflammation and impaired mucosal immunity.^19^ Smad3 has been shown to play an important role in mediating TGF-β signals in T cells, neutrophils and monocytes, and has a role in mediating antiproliferative effects of TGF-β as well as immunomodulatory effects such as the regulation of cytokine production.^19^–^21^ The loss of Smad3 makes mice resistant to chemically induced skin cancer, which is both stimulated and inhibited by inflammation.^22^ In humans, Smad3 mRNA expression is significantly higher in healthy skin of controls as compared with lesional skin of patients with atopic dermatitis.^23^

In this study, the Smad3 knockout mouse model was used to investigate the *in vivo* role of the TGF-β/Smad3 pathway in CHS. The results show that in this murine model Smad3 regulates the allergic inflammation by modifying the expression of cytokines and chemokines, as well as the infiltration of local inflammatory cells.

## Materials and methods

### Mice and sensitization

Smad3^ex8/ex8^ knockout mice of C57BL6 background were kindly provided by Dr Chuxia Deng (NIH, Bethesda, MD, U.S.A.) and bred in our facilities. The resulting progeny were screened by polymerase chain reaction (PCR) to identify Smad3−/− and wild-type (WT) mice. The mice were kept under pathogen-free conditions. All procedures performed were in accordance with instructions and permissions of the Health Services of the State Provincial Office of Southern Finland.

Male or female mice aged 7–10 weeks (age- and sex-matched within each experiment; *n*=5–11 mice per group) were topically sensitized to oxazolone according to the method described by Lauerma *et al.*^24^ The mice were lightly anaesthetized by inhaled Isofluran (Abbott Laboratories, Abbott Park, IL, U.S.A.), their back skin shaved (4 cm^2^), tape-stripped three times, and sensitized to oxazolone (50 μL, 10 mg mL^−1^) in a 4 : 1 acetone : olive oil solution on day 0. A control group (*n*=3–12 mice per group) was treated similarly with vehicle only. On day 7, oxazolone was re-applied on the back skin (50 μL, 1 mg mL^−1^) and the ear pinnae (25 μL, 1 mg mL^−1^). The resulting thickness of the antigen-challenged ears was measured using a micrometer (Mitutoyo, Kanagawa, Japan), before challenge and at 24 and 48 h after challenge. Increased ear thickness is expressed as mean ± SEM. The mice were killed at 48 h and ear draining lymph nodes, ear pinnae and skin biopsies were collected for further analysis. Ear pinnae of approximately equal area were weighed, expressed as mean ± SEM. All experiments were repeated twice.

### Histological analysis and immunohistochemistry

Skin biopsies from allergen-exposed areas obtained at 48 h postexposure were fixed in 10% buffered formalin, embedded in paraffin and cut into 4 μm thick sections. The skin sections were stained with haematoxylin and eosin and toluidine blue, and examined for lymphocytes, eosinophils and neutrophils under light microscopy. Inflammatory cell types were counted in 15 high-power fields at × 1000 magnification and expressed as cells per high-power field.

Frozen, allergen-exposed ear pinnae were stored at −80 °C and embedded in OCT compound until sectioned, fixed in cold acetone, and peroxidase stained with rat antimouse CD3 antibody (Ab) (clone 17A2), rat antimouse CD4 Ab (clone RM4-5) purchased from BD Pharmingen (San Diego, CA, U.S.A.) and with rat antimouse Foxp3 (clone FJK-16s) purchased from eBioscience (San Diego, CA, U.S.A.). Biotin-conjugated secondary Ab antirat IgG (H + L) was purchased from Vector Laboratories (Burlingame, CA, U.S.A.).

### Proliferation test

To reflect lymph node activity *in vivo*, lymph node cell proliferation was measured with no stimulating antigen immediately after isolation. Lymph node cells were seeded at 1 × 10^5^ cells per well, suspended in complete RPMI-1640 medium with Glutamax (Invitrogen Life Technologies, San Diego, CA, U.S.A.) in 96-well plates, after which 1 μCi [^3^H] thymidine per well was added. After 36 h of incubation at 37 °C/5% CO_2_, incorporated radioactivity was determined with a liquid scintillation counter (Trilux 1450 Microbeta; Wallac, Turku, Finland). Results are expressed as mean counts per minute of triplicate wells.

### RNA isolation and cDNA synthesis

Skin biopsies and ear draining lymph nodes were collected at 48 h after allergen challenge. The tissue samples were immediately snap frozen on dry ice and kept at −80 °C until homogenized with an Ultra-Turrax T8 (IKA Labortechnik, Staufen, Germany) in TRIzol (Invitrogen Life Technologies). RNA extraction was performed according to TRIzol instructions, followed by DNAseI (RNase-free; Invitrogen Life Technologies) treatment to remove contaminating genomic DNA, and further extraction with phenol-chloroform-isoamylalcohol (25 : 24 : 1). One microgram of extracted RNA was reverse transcribed into cDNA using MultiScribe reverse transcriptase and random hexamers (Applied Biosystems, Foster City, CA, U.S.A.).

### Real-time quantitative polymerase chain reaction

Quantitative real-time PCR (TaqMan) analysis was performed with ABI Prism 7700 Sequence Detector System (Applied Biosystems) as described previously.^25^ Primers and target-specific probes were purchased as predeveloped reagents [IL-1β, IL-6, tumour necrosis factor (TNF)-α, IL-4, IL-17, IL-10, Foxp3, CXCL9/MIG, CXCL10/IP10, CCL3/MIP-1α, CXCL5/LIX] from Applied Biosystems, or were designed [interferon (IFN)-γ, TGF-β, CCL24/eotaxin-2, CCR3] as described previously.^26^ Endogenous 18S rRNA was used to normalize the gene expression between different samples.

### Statistical analysis

The data were analysed with GraphPadPrism software (GraphPad, San Diego, CA, U.S.A.) and the Mann–Whitney *U*-test was used to compare mouse groups. The data are expressed as mean ± SEM, and *P*<0·05 was considered statistically significant.

## Results

### Topical exposure to oxazolone induces enhanced infiltration of neutrophils in the skin of Smad3-deficient mice

**Fig 1:**
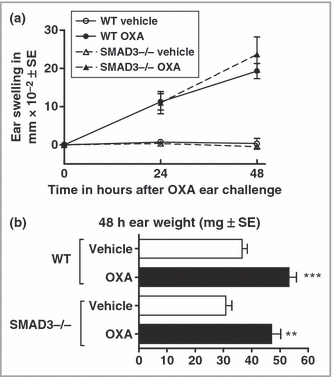
Ear swelling responses after exposure to oxazolone (OXA). Topical application of OXA to the ears induced significant ear swelling in wild-type (WT) and Smad3−/− mice. The swelling of ears was measured at 0, 24 and 48 h after topical exposure to OXA (a), and ear auricles were weighed at 48 h postexposure (b). The results are shown as mean ± SEM. **P<0·01, ***P<0·001.

There was a significant ear swelling response both in WT and Smad3−/− mice that were exposed to oxazolone, measured at 24 and 48 h postallergen exposure (Fig. 1a). Also the weight of ear pinnae increased after allergen exposure (Fig. 1b). The magnitude of the swelling of ears in Smad3−/− mice did not differ from that in WT mice.

**Fig 2:**
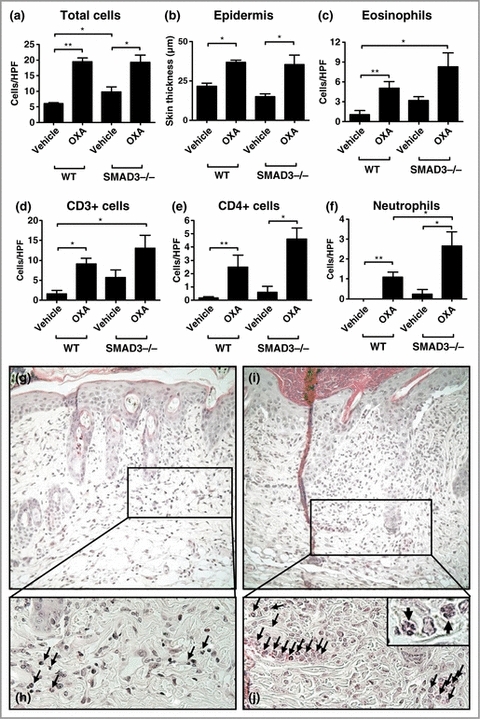
Histological changes in the skin after exposure to oxazolone (OXA). The number of inflammatory cells (a) and the thickness of epidermis (b) increased significantly in the skin after topical exposure to OXA in wild-type (WT) and Smad3−/− mice. Also eosinophils (c), CD3+ (d) and CD4+ (e) cells and neutrophils (f) increased during the contact hypersensitivity response. HPF, high-power field. Haematoxylin and eosin-stained skin sections show the significantly increased infiltration of neutrophils (arrows) in Smad3−/− mice (i, j) (original magnification × 200, × 400, inset × 1000) compared with WT (g, h) (original magnification × 200, × 400). The numbers of CD3+ and CD4+ cells were determined by immunohistochemical analysis. The results are shown as mean ± SEM. *P<0·05, **P<0·01.

Topical exposure to oxazolone induced a delayed-type inflammation in the mouse skin, with increased infiltration of inflammatory cells (Fig. 2a). Eosinophils (Fig. 2c) and T lymphocytes (CD3+, CD4+ cells; Fig. 2d, e) were significantly increased in oxazolone-exposed skin sites both in Smad3−/− and WT mice, with a tendency towards increased numbers of these cells in Smad3−/− mice. The number of neutrophils was significantly higher in oxazolone-exposed skin sites in Smad3−/− mice (Fig. 2f, i, j) compared with WT mice (Fig. 2f–h).

### Proinflammatory, Th2 and regulatory cytokines are increased in oxazolone-exposed Smad3-deficient skin

**Fig 3:**
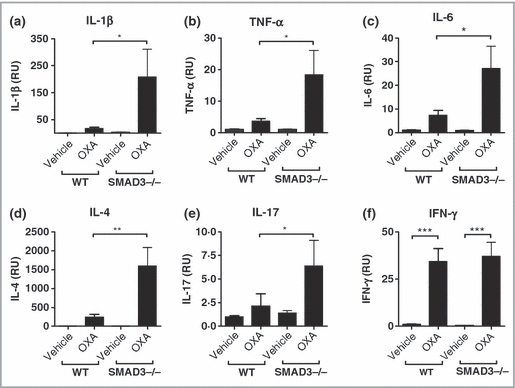
mRNA expression of cytokines in the skin after topical exposure to oxazolone (OXA). mRNA of proinflammatory cytokines interleukin (IL)-1β, tumour necrosis factor (TNF)-α and IL-6 was significantly increased in OXA-exposed skin sites of Smad3−/− mice (a–c) compared with wild-type (WT), as well as IL-4 and IL-17 mRNA (d, e). The expression of interferon (IFN)-γ mRNA during the contact hypersensitivity response to OXA was not modulated by the lack of Smad3 (f). The results are shown as mean ± SEM. *P<0·05, **P<0·01, ***P<0·001.

mRNA of proinflammatory cytokines TNF-α and IL-1β was significantly increased in oxazolone-exposed skin samples of Smad3-deficient mice, compared with WT mice (Fig. 3a, b). The expression of IL-6, an important activator of T cells and mediator of acute phase reactions, was also significantly increased at the mRNA level in Smad3-deficient mice (Fig. 3c).

The expression of Th2 cytokine IL-4 mRNA (Fig. 3d), as well as that of Th17 cell-derived cytokine IL-17 (Fig. 3e), were significantly increased in oxazolone-exposed skin of Smad3-deficient mice compared with their WT siblings. In contrast, the major Th1 cytokine, IFN-γ, was unaltered by the lack of Smad3 (Fig. 3f).

**Fig 4:**
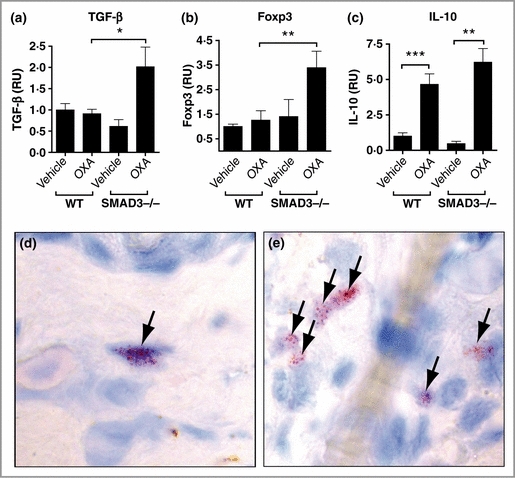
mRNA expression of interleukin (IL)-10, transforming growth factor (TGF)-β and Foxp3, and Foxp3+ cells in the skin after topical exposure to oxazolone (OXA). The expression of TGF-β mRNA and Foxp3 mRNA was significantly increased in Smad3−/− mice compared with wild-type (WT) (a, b). The expression of IL-10 mRNA in the skin was not affected by the lack of Smad3 (c). Immunohistochemical analysis of Foxp3+ cells (arrows) in OXA-treated skin in WT (d) and Smad3−/− mice (e). (a–c) The results are shown as mean ± SEM. *P<0·05, **P<0·01, ***P<0·001.

The mRNA expression of TGF-β was significantly increased in Smad3-deficient mice compared with WT mice after oxazolone treatment (Fig. 4a). Also the expression of Treg-associated Foxp3 mRNA was markedly increased in Smad3−/− mice (Fig. 4b). In line with the results at the mRNA level, immunohistochemical staining showed increased numbers of Foxp3+ cells in the skin in Smad3−/− mice (Fig. 4d, e). The expression of IL-10 mRNA, an important regulatory cytokine,^27^,^28^ was not affected by the lack of Smad3 (Fig. 4c).

### Cytokine profiles in draining lymph nodes differ from the local response in the skin

**Fig 5:**
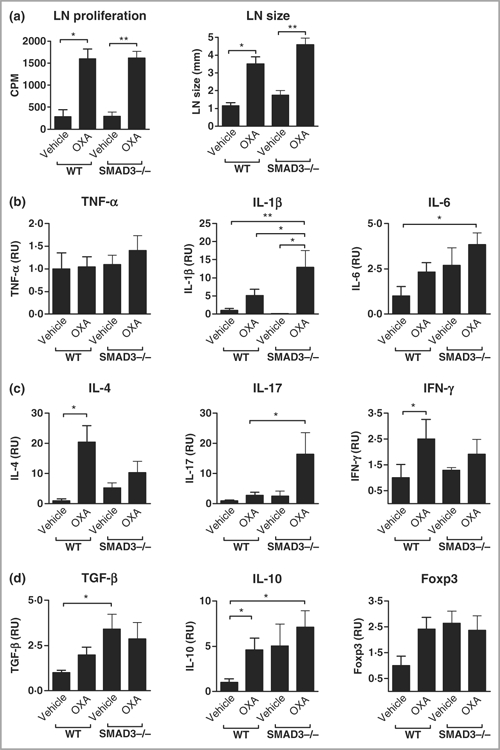
Lymph node (LN) size, proliferation, and mRNA expression of cytokines and Foxp3. LN cell proliferation and size (a) were increased in oxazolone (OXA)-exposed mice. The expression of interleukin (IL)-1β (b) and IL-17 (c) mRNA was significantly increased in Smad3−/− lymph nodes after exposure to OXA, compared with wild-type (WT), while that of tumour necrosis factor (TNF)-α, IL-6, IL-4 and interferon (IFN)-γ was not (b, c). Transforming growth factor (TGF)-β mRNA expression in the LNs of vehicle-treated mice was significantly higher in Smad3−/− mice (d). The results are shown as mean ± SEM. *P<0·05, **P<0·01.

Ear draining lymph nodes were significantly larger in mice that were exposed to oxazolone, and the proliferation rates of lymph node cells from the oxazolone-exposed groups were approximately fivefold higher compared with control groups. Lymph node size and rate of lymph node cell proliferation did not differ between Smad3−/− mice and WT mice (Fig. 5a).

In the lymph nodes the pattern of IL-1β and IL-17 mRNA expression was similar to that in the skin, i.e. they were expressed at significantly higher levels in Smad3−/− mice compared with WT mice after oxazolone exposure. Further, there was a slight tendency for higher levels of TNF-α, IL-6 and TGF-β mRNA in Smad3−/− mice, although not statistically significant (Fig. 5b–d). IL-4 and IFN-γ mRNA levels, on the contrary, tended to be lower in Smad3−/− mice lymph nodes. In the Smad3−/− control group, the expression of TGF-β mRNA was notably high, and there was also a tendency towards increased levels of IL-10 and Foxp3 mRNA in the lymph nodes, compared with WT control mice (Fig. 5d).

### Expression of neutrophil-attracting and Th2 chemokines is significantly increased in the skin of Smad3-deficient mice

**Fig 6:**
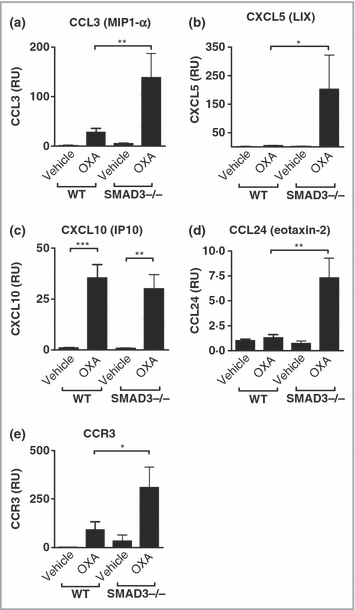
Chemokine and chemokine receptor mRNA expression in the skin after topical exposure to oxazolone (OXA). Neutrophil-attracting chemokines CCL3/MIP-1α (a) and CXCL5/LIX (b) were significantly increased in OXA-treated Smad3−/− mice compared with wild-type (WT). The expression of Th2-associated chemokine CCL24 (eotaxin-2) was significantly increased in OXA-treated Smad3−/− mice compared with WT (d), while that of Th1-related chemokine CXCL10/IP10 was not (c). The expression of Th2-associated chemokine receptor CCR3 was significantly increased in Smad3−/− mice compared with WT (e). The results are shown as mean ± SEM. *P<0·05, **P<0·01, ***P<0·001.

The mRNA expression of neutrophil-attracting chemokines CCL3 and CXCL5 was significantly increased in oxazolone-treated Smad3−/− mice compared with WT controls (Fig. 6a, b). The expression of Th1-related chemokine CXCL10/IP10 was unaltered by the lack of Smad3 in the skin of mice that were exposed to oxazolone (Fig. 6c). Similarly, there was no difference in the expression of CXCL9/MIG, another Th1-related chemokine (data not shown). However, the expression of Th2-associated chemokine CCL24 (eotaxin-2) was significantly increased in Smad3-deficient mice compared with WT mice (Fig. 6d). Further, the expression of CCR3, a receptor for CCL24, was also significantly increased in Smad3−/− mice when compared with WT controls (Fig. 6e).

## Discussion

Targeted disruption of Smad3 in mice results in immune dysregulation and susceptibility to opportunistic infection. Smad3-deficient T cells show an activated phenotype *in vivo*, and reduced responsiveness to TGF-β*in vitro*.^19^ It has also been demonstrated that Smad3-null splenocytes have defects in TGF-β-mediated repression of cytokine production.^21^ In various disease models, in the lack of Smad3, wound healing was accelerated,^20^ the fibrotic response to bleomycin in mouse lungs was attenuated^29^ and our laboratory has recently shown that the response to topical exposure to ovalbumin is diminished when Smad3 is lacking in the mouse atopic dermatitis model.^30^ In the present study, Smad3 deficiency greatly increased the expression of proinflammatory, Th2 and Th17 type cytokines and chemokines and regulatory components such as TGF-β and Foxp3 in the skin, as well as neutrophil infiltration during the CHS response to oxazolone.

Proinflammatory cytokines are upregulated to a certain degree in local trauma. Stimulation with proinflammatory cytokines induces adhesion molecules,^31^ which facilitate, or are required for, optimal skin sensitization.^4^ According to previous studies, TGF-β secretion inhibits the production of inflammatory cytokines and chemokines in macrophages, including IL-1β, TNF-α and IL-8.^32^ Conversely, Smad3 deficiency renders immune cells insensitive to such inhibition.^21^ We observed significantly increased production of proinflammatory cytokines IL-6, IL-1β and TNF-α at the mRNA level in skin sites that were exposed to oxazolone in the Smad3-deficient mice. An increased expression of inflammatory cytokines was also detectable in the draining lymph nodes of the Smad3−/− mice, although at less striking levels than in the skin (Fig. 5b).

The general concept is that CHS is mediated by IFN-γ-producing CD8+ T cells, while IL-2-, IL-4- and IL-10-producing CD4+ T cells regulate the response. There are, however, results that also indicate an effector role for CD4+ T cells in the CHS response to oxazolone in C57BL6 mice.^33^ In addition, the Th2 type cytokine IL-4 might also have a proinflammatory role, as shown by impaired CHS responses in mice lacking IL-4.^7^ In this study, the level of IL-4 mRNA in the skin of Smad3−/− mice was strongly increased after exposure to oxazolone, while the expression of IFN-γ remained at the same level in Smad3−/− mice as in their WT siblings (Fig. 3d, f). Thus, IFN-γ production by, presumably, mainly CD8+ T cells in this model^33^ is not affected by the lack of Smad3. In contrast, the production of IL-4, mainly by CD4+ T cells, according to previous findings,^33^ is strongly modulated by the lack of Smad3.

Several studies have shown that TGF-β can inhibit both Th1 and Th2 differentiation *in vitro*, and that Th2 differentiation is more sensitive to inhibition by TGF-β.^9^ Studies with Smad3-deficient immune cells have shown that Smad3−/− T cells have an activated phenotype *in vivo*, and that Smad3−/− spleen cells have defective TGF-β-mediated inhibition of cytokine production *in vitro*.^19^,^21^ Thus, the significantly increased production of IL-4 mRNA in Smad3−/− skin after oxazolone exposure might be a consequence of reduced inhibition of IL-4 production by TGF-β, or increased conversion of CD4+ T cells into IL-4-producing Th2 cells. Moreover, there was a slight tendency towards increased numbers of CD3+ and CD4+ cells in the skin of Smad3−/− mice, which also could contribute to the increased expression of IL-4. Nevertheless, the expression of high levels of IL-4 mRNA after oxazolone exposure was restricted to the skin and was not detected in the lymph nodes in Smad3−/− mice.

IL-1β, IL-6 and TGF-β are essential for the differentiation of Th17 cells in mice.^14^ After exposure to oxazolone, all three of them were significantly increased at the mRNA level in the skin in Smad3−/− mice, and they were also abundant in lymph nodes. We consequently found increased levels of IL-17, product of Th17 cells, both in the skin and in the lymph nodes of Smad3−/− mice during the CHS response to oxazolone. IL-17 is important in CHS, as demonstrated by reduced CHS in mice that lack IL-17.^34^ Hapten primed T cells produce IL-17, and in particular a CD8+ subpopulation of T cells that produce IL-17 has been demonstrated as important for the elicitation of CHS responses.^13^ Our results suggest that Smad3 is involved in the regulation of IL-17 in the CHS response to oxazolone.

TGF-β plays a crucial role in skin-induced tolerance and is important in the induction of Tregs that inhibit CHS.^35^ TGF-β is needed to maintain normal peripheral Treg numbers^36^ and TGF-β signalling enhances the conversion of naive T cells into Tregs.^37^ Although Smad3 has recently been implicated as an important enhancer of Foxp3 expression,^38^ in our study the lack of Smad3 in knockout mice did not result in reduced, but rather increased expression of Foxp3 mRNA and elevated numbers of Foxp3+ cells in the skin during the CHS response to oxazolone. These results suggest there are other than Smad3-dependent pathways of Foxp3 induction at the site of inflammation. Our results also indicate that IL-10 is not involved in that pathway. Notably, in the lymph nodes of Smad3−/− control mice, there was a high level of TGF-β expression, and a tendency for increased expression of IL-10 and Foxp3 (Fig. 5b–d) compared with WT, suggesting increased regulatory activity in general in mice that lack Smad3.

The CCR3 receptor is expressed predominately by eosinophils, and to a smaller extent by basophils and Th2 polarized lymphocytes,^39^ which are all relevant to allergic inflammation. CCR3 ligands, such as the Th2-associated CCL24/eotaxin-2, amplify the Th2 response in allergic inflammation. In this study, the mRNA of both CCR3 and CCL24 was significantly elevated in the skin of oxazolone-challenged Smad3−/− mice, which may reflect the slight (although not statistically significant) increase of eosinophil infiltration in the skin of Smad3−/− mice.

During acute inflammation, an initial infiltration of neutrophils characterizes the leucocyte recruitment, being later replaced by monocytes, lymphocytes and other inflammatory cells.^40^ According to earlier studies, Smad3 deficiency impairs the chemotactic response of neutrophils to TGF-β*in vitro*.^19^ Accordingly, in wound healing experiments, both neutrophils and monocytes were largely absent in mice lacking Smad3.^20^ In contrast to these findings, we observed that during the CHS response to oxazolone there is a significantly increased neutrophil infiltration into the skin of mice that lack Smad3. We also analysed the expression levels of chemokines CCL3/MIP-1α and CXCL5/LIX, which are both involved in neutrophil recruitment,^41^–^47^ and both of these chemokines were significantly increased at the mRNA level in oxazolone-exposed skin of Smad3−/− mice. It has been demonstrated that TGF-β inhibits CCL3 expression, mediated via Smad3 in macrophages *in vitro*,^48^ which might explain our observations of increased CCL3 as well as CXCL5 during the CHS response in Smad3−/− mice. The increased levels of attracting chemokines (CCL3 and CXCL5), in turn, might bring about the increased infiltration of neutrophils into the skin. Evidence is also accumulating that IL-17, which was significantly increased in Smad3-deficient lymph nodes and skin during the oxazolone response, significantly stimulates neutrophil maturation, migration and function.^49^

In conclusion, our results suggest that Smad3-mediated signalling pathways play an important part in the CHS response to oxazolone. Complete loss of this signalling intermediate resulted in accelerated expression of proinflammatory, Th17 and Th2 type cytokines in the skin, as well as increased infiltration of neutrophils to the site of inflammation. Moreover, the lack of Smad3 in knockout mice did not result in reduced, but rather increased expression of Foxp3 mRNA and elevated numbers of Foxp3+ cells in the skin during the CHS response. Unravelling the mechanisms in TGF-β-mediated regulation of CHS may provide insights for future prevention and treatment modalities in this often disabling disease.
